# FlexPepDock lessons from CAPRI peptide–protein rounds and suggested new criteria for assessment of model quality and utility

**DOI:** 10.1002/prot.25230

**Published:** 2017-02-16

**Authors:** Orly Marcu, Emma‐Joy Dodson, Nawsad Alam, Michal Sperber, Dima Kozakov, Marc F. Lensink, Ora Schueler‐Furman

**Affiliations:** ^1^ Department of Microbiology and Molecular Genetics Institute for Medical Research Israel–Canada, Faculty of Medicine, the Hebrew University of Jerusalem Israel; ^2^ Department of Applied Mathematics and Statistics Stony Brooks University Stony Brook New York 11794; ^3^ University of Lille, CNRS UMR8576 UGSF Lille 59000 France

**Keywords:** Peptide docking, FlexPepDock, CAPRI, model assessment, impact of low-accuracy models, binding hotspots

## Abstract

CAPRI rounds 28 and 29 included, for the first time, peptide‐receptor targets of three different systems, reflecting increased appreciation of the importance of peptide‐protein interactions. The CAPRI rounds allowed us to objectively assess the performance of Rosetta FlexPepDock, one of the first protocols to explicitly include peptide flexibility in docking, accounting for peptide conformational changes upon binding. We discuss here successes and challenges in modeling these targets: we obtain top‐performing, high‐resolution models of the peptide motif for cases with known binding sites but there is a need for better modeling of flanking regions, as well as better selection criteria, in particular for unknown binding sites. These rounds have also provided us the opportunity to reassess the success criteria, to better reflect the quality of a peptide‐protein complex model. Using all models submitted to CAPRI, we analyze the correlation between current classification criteria and the ability to retrieve critical interface features, such as hydrogen bonds and hotspots. We find that loosening the backbone (and ligand) RMSD threshold, together with a restriction on the side chain RMSD measure, allows us to improve the selection of high‐accuracy models. We also suggest a new measure to assess interface hydrogen bond recovery, which is not assessed by the current CAPRI criteria. Finally, we find that surprisingly much can be learned from rather inaccurate models about binding hotspots, suggesting that the current status of peptide–protein docking methods, as reflected by the submitted CAPRI models, can already have a significant impact on our understanding of protein interactions. Proteins 2017; 85:445–462. © 2016 The Authors Proteins: Structure, Function, and Bioinformatics Published by Wiley Periodicals, Inc.

## INTRODUCTION

The link between form and function has made clear the usefulness of high‐resolution 3D protein structures in determining the details of biological processes. Once the structure of a protein is known, residues critical for stability, as well as its active sites can often be located, and targeted mutations can be introduced. Similar structures can be identified and used to infer about the studied protein.^1^ Similarly, resolution of protein complexes can provide valuable information concerning the interaction mechanisms of proteins, allowing for the improved characterization and manipulation of signaling pathways in the cell. Once the details of the interface are known, inhibitors and activators of the target proteins can be designed based on features of the binding interface.^2^, ^3^

Despite the high demand for crystallography‐ and NMR‐derived structures, only a small percentage of proteins, and even less protein complexes, have been solved (as of 9/2016: 113,000 protein structures, among them 5000 complexes; see the Protein Data Bank, PDB, at www.rcsb.org 4). As a result, much focus has been placed on developing computational algorithms for the formulation of structural models of proteins and protein complexes. Over the past two decades, the community‐wide experiment for the blind prediction and assessment of protein structures, CASP (critical assessment of structure prediction), has considerably spurred the development of ever improving and impressive tools for structure prediction, involving increasingly better models, more participating groups and different algorithms and approaches to solve the challenge.^5^ As a parallel to CASP, the CAPRI (Critical Assessment of PRotein Interactions) experiment has spurred development of docking protocols that will generate a structure of a complex starting from the monomer structures^6^, ^7^ (for assessment of performance at the 6th CAPRI meeting in Tel Aviv, see publications in this edition of the *Proteins* journal). As CAPRI (and CASP) evolved, new types of challenges have been defined and put up to further increase the applicability of computational models to the characterization of the structure of proteins and their interactions. This has led to the adaptation of existing and the development of new tools to new applications (e.g., the docking of proteins to biomolecules such as RNA^8^ and sugars,^9^ the prediction of water molecules at interfaces,^10^ the prediction of effect of mutations on binding affinities,^11^ the identification of successful interface designs,^12^ and many others).

Peptide‐protein interactions have gained significant attention as crucial players in cellular regulation. Thus, the accurate modeling of these interactions, which involve the binding of a short, linear stretch that often contains a characterized sequence motif (alone, or within the context of a usually unstructured region in a larger protein), is of primary importance. The intrinsic flexibility of short peptides makes this type of interaction particularly difficult to model, as in contrast to many protein–protein interactions mediated by structured domains, the many degrees of freedom of the peptide need to be taken into account during the docking process. Our peptide docking protocol, Rosetta FlexPepDock, was one of the first to explicitly allow for the sampling of full peptide conformational flexibility during the docking process, by adapting RosettaDock to include peptide backbone degrees of freedom in the Monte‐Carlo sampling process.^13^, ^14^ Rosetta FlexPepDock refinement can refine an initial model of an interaction (up to 3.0–5.0 Å peptide backbone RMSD away) to high resolution. Given a binding site (known, or predicted using, e.g., solvent mapping as in our PeptiMap protocol^15^), the corresponding *ab initio* FlexPepDock version can fold the peptide within the binding site using the Rosetta fragment‐based approach. In our new PeptiDock protocol, we have taken this approach one step further to allow full *ab initio* docking without prior knowledge of the binding site: for peptides with known binding motif (extracted, e.g., from the ELM database of eukaryotic linear motifs, elm.eu.org,^16^ or from the literature), we extract fragments from the PDB based on this sequence motif, and map these fragments using the PIPER^17^ rigid body docking protocol (instead of mapping solvent molecules, as e.g. in PeptiMap^15^). The pooled results are then clustered, and an approximate model (within 4.0 Å RMSD) can usually be found among the top‐ranking clusters (*to be published)*.

Since then, a range of other original and very successful tools for modeling peptide‐protein complexes have been proposed,^18^, ^19^, ^20^, ^21^, ^22^, ^23^, ^24^, ^25^, ^26^ and a book about modeling these interactions is about to appear (Modeling Peptide–Protein Interactions, to appear in the Methods in Molecular Biology Series, Ed. Springer). This progress has been recently spurred also thanks to the addition of this type of interactions to the CAPRI pool of challenges (see other manuscripts in this editions of Proteins). With the definition of a new type of challenge comes the need to redefine measures of success. CAPRI criteria for the quality of a model focus on ligand and interface Root‐Mean‐Square Deviation (L‐ and I‐RMSD) measurements, along with cross‐interface residue contact recovery (fnat).^27^, ^28^ These measures have been adjusted ad hoc to better reflect the quality of peptide‐protein interactions (Table 1). The success of any applicative modeling endeavors is contingent upon the quality of the models produced and, consequently, on the criteria used to define high‐quality models. In general, the success of a model is determined by how closely it aligns to a solved crystal structure of the same protein. Whether using homology‐based approaches or *de novo* techniques, protein models can now be generated with impressive accuracy. There is, of course, much room for improvement, and many research groups are forever seeking ways to advance their protocols. This begs the question: at what point is “pretty good” good enough? Could it be that success might not be measured in sub‐Ångström RMSD values? When is a model capable of telling us as much as we need to know to make our binding predictions and design our potential drugs? Conversely, are there critical features that are missed by the currently established criteria for model quality assessment?

**Table 1:** Current CAPRI Success Criteria for Peptide–Protein Docking (Modified From^27^, ^28^)

(A) Measures	
Interface residues	<8.0 Å between any two CB atoms (CA for Gly) across interface
Native contacts	<4.0 Å between any two atoms across the interface (residue‐based)
Clashes	<3.0 Å between any two atoms across the interface (atom‐based)
(B) Classification	
High quality (H)	fnat [0.8 . 1.0] & (L‐RMSD ≤ 1.0 Å ǁ I‐RMSD ≤ 0.5 Å)
Medium (M)	fnat [0.5 . 0.8] & (L‐RMSD ≤ 2.0 Å ǁ I‐RMSD ≤ 1.0 Å) ǁfnat [0.8 . 1.0] & (L‐RMSD > 1.0 Å & I‐RMSD > 0.5 Å)
Acceptable (A)	fnat [0.2 . 0.5] & (L‐RMSD ≤ 4.0 Å ǁ I‐RMSD ≤ 2.0 Å) ǁfnat [0.5 . 1.0] & (L‐RMSD > 2.0 Å & I‐RMSD > 1.0 Å)
Incorrect (I)	The rest

Accuracy criteria thus depend primarily on practical use, that is, how a protein complex structure is used for further study. Arguably, major applications involve the identification of critical features at the interface, such as specific hydrogen bonds, salt bridges and interactions that involve aromatic rings that are responsible for binding affinity and specificity, which can then be reinforced or targeted in design studies.^2^ These might necessitate accurate measures, for example, the correct positioning of side chains at the interface. Beyond this, a popular use of complex structures is to close in on the few critical interface hotspots,^3^, ^29^ whose mutation would abolish the interaction. Energy‐based approaches for computational alanine scanning aim to estimate change in binding affinity, ΔΔ*G*, by comparison of the energetics of the wild‐type and the modeled alanine‐substituted structure (examples include implementations by molecular modeling tools such as FoldX,^30^ mmPBSA,^31^ and Rosetta^32^). In addition, machine‐learning approaches compile a range of spatial and other features to train predictors and classifiers, to assess the effect of mutation on the strength of binding, similar in line to the prediction of effects of mutation on protein monomer stability.^33^ However, despite considerable advances in this field, the correlation between prediction and experiment on independent validation sets has remained disappointingly low, often barely crossing random performance.^12^, ^34^, ^35^ Many reasons are to blame, not least the fact that effects on binding affinity measured for the same mutation in two independent experiments are also rather weakly correlated (*R* = 0.7^30^), raising doubts on the general reliability and reproducibility of binding experiments on the one hand, and concerns related to overfitting of predictors on the other.^36^ With this variability of predictions based on crystal structures, it is therefore not clear which measure would identify successful models for hotspot identification. Despite these reservations, it is apparent that a solved complex structure is of fundamental relevance for the identification of both critical features at the interface, as well as hotspots.

This study consists of two parts: we first present the performance of Rosetta FlexPepDock in the peptide‐docking rounds of CAPRI. Here, we highlight successes that profile our protocol as very accurate and top‐performing, in particular in the modeling of the peptide motif region onto the receptor in cases where the binding site is known (Targets T60‐64—nuclear localization motifs bound to the minor site of importin, and T67—WW domains bound to PPXY motifs). We also identify challenges, in particular the selection of successful receptor structure templates and the necessity of robust protocols for the selection, for example of a receptor template and a binding site, and suggest how these can be attacked to further improve modeling.

The second aim is to assess, on the example of peptide‐protein interactions, how accurate structural models of interactions need to be to provide information similar to an experimentally determined structure for practical use. We inspect models of peptide‐protein complexes of varying accuracies, submitted by several anonymous groups in recent CAPRI competitions (i.e., Targets 60–64 of round 28, Targets 65–67 of round 29), and investigate the connection between accuracy, as measured in the CAPRI experiment, and their practical use. We assess the ability of these models to capture the details of an interface observed in the solved crystal structure (e.g., specific hydrogen bonds, summarized in Tables 2, 3, 4), and suggest two additional measures that provide complementary information to the current criteria, which can be used to refine the definition of model accuracy: S‐RMSD—interface side‐chain RMSD, and *f*nat_hb,_—the fraction of native hydrogen bonds recovered by a model. In addition, we also assess the models for their ability to reveal known interface hotspots. For this, we characterize each of the models using a representative set of predictor programs designed to detect interface hotspots, namely Rosetta alanine scanning (as implemented in the Robetta alanine scanning server^32^), FoldX^30^ (version 2.5), and mCSM^33^ (one of the best‐performing machine‐learning tools that is based on spatial signatures of amino acid residues around a tested hotspot residue). We show that even models classified as poor (i.e., incorrect or acceptable quality) can be surprisingly useful for certain applications. Thus, the quest for ever improved modeling tools results both in the generation of high‐accuracy models, but also provides a plethora of more approximate models, and all together will significantly enhance our understanding of more and more protein interactions.

**Table 2:** Residues Involved in the Peptide–Receptor Interactions for CAPRI Targets T60‐64: Importin α − NLS of RNA Helicase Guα + Peptides Derived from mRNA Display (PDB ids 3zin, 3zio, 3zip, 3ziq, 3zir)^39^

Peptide residue^a^	Contacting receptor residues^b^	Additional information
Ser P_−3’_(W,R)	S406 (HB:SM)^c^, G407	
Arg P_−2'_ (I,A,S,V)	D325 (SB), A364 (HB:SM), G365, R366, S406, G407	
Gly P_−1'_ (H/Q)	A364, N403 (HB:MS), S406, G407	
Gln P_0'_ (R)	A364, W399, N403 (HB:MS)	
**Lys P_**1'**_**	*V321* d (HB:SM), *T322*, *G323*, *T324*, *T328* (HB:SS), *N361* (HB:SM), *G365*	[K‐>R: <5% NLS‐GFP import]^37^
**Arg P_**2'**_**	*T322*, *W357* (HB:MS), *S360* (HB:SS), *N361* (HB:MSx2), ***E396*** (**SB**), *W399* (+π;HB:MS), *N403*	[R‐>A: reduction in NLS‐GFP import]^38^; [R‐>A/K: <5% NLS‐GFP import]^37^
		[E396R: 100x less binding (E402)]^39^ ^e^; [E396Q: reduced binding of bi‐partite NLS‐GFP]^40^ ^f^
Ser P_3'_ (G,T,K)	W357	
**Phe P_**4'**_** (aro)	***R315*** (π+), *E354*, *W357* (ππ)	[W‐>V: <5% NLS‐GFP import in mouse importin]^37^
		[R315A & Y277A: 10x less binding of bipartite NLS‐GFP (R321, Y283)]^40^
Ser P_5’_	–	
Lys (+) P_6’_	–	
**Ala P_**7’**_**	*W357*, ***E396***	
**Phe P_**8’**_**	*K353* (π+), *W357*, ***E396***	[F‐>A: <5% NLS‐GFP cargo import]^37^
Gly P_9’_	–	

a In parentheses: amino acids in other peptides (T61–T64), if not identical. +: basic residue; aro: aromatic residue.

b Native contact between peptide and receptor residues are defined as by CAPRI: at least one atom pair across the interface within 4.0 Å distance.

c Specific interactions with the peptide are indicated in parentheses next to the receptor residue: SB: salt bridge; HB: Hydrogen bond between peptide and receptor (involving side‐chain, S or main chain, M); +π: cation −π . Hydrogen bonds and Salt bridges are identified according to HBplus, see Methods.

d Residues with conserved interactions are in italics.

Residue numbering in respective studies is indicated in parentheses.

Note that effect is observed only at 37°C, not at 25°C.

Highlighted in bold are the peptide residues that are part of the binding motif (first column), and the receptor residues for which experimental information on their contribution to binding is available (detailed in the last column).

**Table 3:** Residues Involved in the Peptide–Receptor Interactions for CAPRI Target T67: Nedd4 Third WW Domain–ARRDC3 PY1 (PDB id 4n7h)^41^ (Legend as in Table II

Peptide residue	Contacting receptor residues	Additional information
Glu P_−2’_	**W449** (HB:MS)	
Ala P_−1’_	**W449**	
**Pro P_**0’**_**	F438, T447, T448, ***W449***	[WWOX WW1‐ErbB4 PY3] P‐>A: NBD^42^ ^a^ [Yap2 WW1‐ErbB4 PY3] P‐>A: NBD^43^ [WWOX WW1 domain ‐ WBP PY3] W449Y: binder ‐> NBD (W44)^44^ [WWOX WW2 domain ‐ WBP PY3] E430R+ Y449W: NBD‐>binder (E66,Y85)^44^
**Pro P_**1’**_**	A432, P433, F438, *T447* (HB:MS), W449	[WWOX WW1‐ErbB4 PY3] P‐>A: NBD^42^ [Yap2 WW1‐ErbB4 PY3] P‐>A: NBD^43^
Ser P_2’_	T447	
**Tyr P_**3’**_**	I440, D441, *H442* (HB:SS), K445, T446, T447	[WWOX WW1‐ErbB4 PY3] P‐>A: NBD^42^ [Yap2 WW1‐ErbB4 PY3] P‐>A: NBD^43^
Ala P_+1,_ Glu P_+2_	–	
**Val P_**+3**_**	E428, **R430**, F438, I440, H442, T447	V‐>I: ∼2x less binding^41^ [WWOX WW1 domain ‐ WBP PY3] R430A: binder ‐> NBD (R25)^44^

^g^NBD—no binding detected.

Highlighted in bold are the peptide residues that are part of the binding motif (first column), and the receptor residues for which experimental information on their contribution to binding is available (detailed in the last column).

**Table 4:** Residues Involved in the Peptide–Receptor Interactions for CAPRI Targets T65/T66: Bacterial RNase/PriA Helicase—Single Strand Binding Protein (SSB) c‐Terminal Peptide (PDB id 4z0u^45^/4nl8^46^)

	T65		T66
Peptide residue	Contacting receptor residues^a^	Additional information^45^	Contacting receptor residues^b^
ASP P_−3_’	**K3** (HB:MS;SS), Y28(HB:SS), **R29**(SB)	K3A: ND; R29A: 8x less binding	R699 (HB:SS)
ILE P_−2_’	Y28, **R31**	R31A: 10x less binding	S696, R697, V698
**PRO P_**−1**_’**	V5, Y28, A58, L59, **K60** (HB:MM), E61, C63	K60A: 10x less binding	–
**PHE C‐term**	L26, Y28, **R31**, ***K33*** (HB:MS), A58, **K60**	K33A: 3x less binding	V341, R697 (oxt‐SB/HB:MS^c^)

a Chains B–E were used for further evaluation since the A–D complex contains far less interactions. Underlined are interactions that appear only in chains B–E.

b Chains B–D were used.

c HB:F_C’_‐R697 taken from chains E–F.

Highlighted in bold are the peptide residues that are part of the binding motif (first column), and the receptor residues for which experimental information on their contribution to binding is available (detailed in the third column).

## METHODS

### Modeling of peptide–protein complexes in CAPRI rounds 28–29 using Rosetta FlexPepDock

We used our Rosetta FlexPepDock program to generate models of peptide–protein complexes, starting from an approximate model of the interaction (see below). Docking was performed as previously described,^13^, ^14^ using Rosetta version 3.4 with the scoring function score12 (with modifications to the electrostatic potential, as specified in Alam *et al*.^47^), and selecting models using interface score, as well as reweighted score.^47^, ^48^ In short, the structure was first prepacked to remove internal clashes in the receptor (and the peptide), by separating the two partners, repacking each, and putting them back together. Then, the complex was either refined using the FlexPepDock refinement option,^13^ or the peptide was modeled using fragments with the ab initio FlexPepDock protocol.^14^

#### Selection of receptor template structure

For each target, we collected all solved homolog structures of the receptor, and focused in particular on bound conformations (see Results). For T60‐64, we used the structure of importin bound to a major‐groove specific peptide, SV40Tag (PDB id 1ejl^39^), since the peptide was found to bind both the minor and the major site in the crystal. For T67, we proceeded with the solved structure of a homolog receptor bound to a template (PDB id 1eg4^50^), rather than with the structure of the free receptor provided by CAPRI (see Results). For T65, many different structures had been solved of the unbound RNAse, showing a range of loop conformations (see Results for template selection). For T66 only the provided, non‐bound structure was available. When using a template of different sequence (receptor and/or peptide), the sequence of the receptor and the peptide were adjusted using Rosetta fixed backbone design^51^ to replace amino acids at relevant positions.

#### Mapping of binding sites

To identify the potential binding sites on the receptor structures for T65 and T66, we applied our PeptiMap protocol that is based on solvent mapping using FTmap to identify sites particularly suitable for peptides^15^ (as implemented in our server, http://peptimap.bu.edu). For T66, the structure was first decomposed into individual domains, and each was mapped separately (masking regions at domain‐domain interfaces).

For T65 we also tried a new approach, in which we used an adaptation of FTmap to map the receptor with fragments of SSB‐terminal peptide conformations, extracted from structures of solved SSB‐receptor complexes. Those fragments were individually docked using PIPER,^17^ top scoring models (250 from each docking run) were combined together and clustered (with a clustering radius of 4.0 Å backbone RMSD). A representative model of each cluster was further minimized using CHARMM to improve the model quality. This application is a precursor of a more general implementation of a global peptide docking approach, PeptiDock, in which fragments selected based on a known peptide binding motif are mapped to the receptor using PIPER rigid body docking,^17^ to generate an approximate model of a peptide‐receptor interaction (within 4.0 Å RMSD) (See Introduction).

#### Generation of starting models

For T60‐64 and T67, we generated starting models based on homologous interactions. For T65 and T66, we generated starting models by arbitrarily positioning an initial peptide conformation (taken from a structure of SSB bound to another receptor, e.g. PDB id 3sxu^52^) into a given binding site. *Ab initio* FlexPepDock was then applied to generate a final model of the interaction. For the new PeptiDock mapping, a starting structure was already provided for further refinement.

### Assessment of model quality and contribution to our understanding of an interaction

#### New measures for CAPRI assessment

The current CAPRI criteria are listed in Table 1. In addition, we use the Interface side‐chain RMSD (S‐RMSD), routinely calculated in CAPRI in the same way as Interface backbone RMSD (I‐RMSD), but for side chains instead of the main chain atoms. We also include the fraction of native interface hydrogen bonds (*f*nat_hb_): Interface hydrogen bonds (including short range salt bridges) were detected using the program HBplus,^53^ and *f*nat_hb_ was calculated analogously to *f*nat, by counting the fraction of identified native hydrogen bonds in the models.

#### Definition of interface hotspots in targets 60–67

Residues were defined as interface hotspots, if they had been shown by experiment to significantly affect binding upon their mutation, and were reported as motif residues of the peptide. We included both residues tested on the proteins involved in the specific interaction, as well as information on homolog interactions ‐ for known peptide motif and conserved receptor residues (see Tables 2, 3, 4). We note that additional residues could be important, but without experimental evidence we did not include them in our list. Below we detail additional criteria applied to each of the systems. T60‐64 (importin minor groove—NLS peptides): This target concentrated on the binding of peptides to the minor groove on importin.^37^ We distinguished between motif residues that were already known to be critical for NLS binding and new, minor‐groove specific residues that were structurally characterized for the first time in the T60‐64 structures. We note that in this study we have restricted our analyses to the structure of the peptide bound to the *minor* groove, as this is the dominant interaction and contribution. T67 (WW domain‐PPXY interaction): As was done for importin, we also distinguished here between motif residues that had been characterized previously in studies on other proteins and the newly identified important residue that is responsible for binding specificity in the Nedd4‐WW domain ARRCD3‐PPXY motif interactions. Thus, even though peptide V P _+ 3’_ position shows a smaller effect on binding upon mutation, it is important in determination of binding specificity.^41^ For the RNase—SSB peptide interaction (T65),^45^ we included receptor residues with reported strong and weak effects. For this Target we have chosen the complex between chains B:E in the crystal structure, as in this complex the local binding region is best defined according to B‐factor, and the largest number of hydrogen bonds between receptor and peptide is observed. No information was available for T66.

#### Alanine scanning for hotspot identification

The following computational alanine scanning protocols were applied to identify hot‐spots at the interface: (1) Robetta alanine scanning,^32^ as implemented in *http:://robetta.org* (a local executable was provided to us by Tanja Kortemme) and a newer implementation in Rosettascripts.^54^ Both protocols remove the side‐chain of the tested residue, but do not change the surrounding residues at the interface. This approach was found to work best in several large‐scale tests^32^, ^55^; (2) FoldX versions 2.5.2 and 3^30^, ^56^; and (3) mCSM^33^ (using a script provided by David Ascher to run the protocol on many models). All these different protocols were applied to the target crystal structures, and the Robetta, FoldX2.5 and mCSM protocols were selected to scan for hotspots in models. mCSM was used to characterize mutations to amino acids different from alanine. A cutoff of ΔΔ*G* = +0.95 U (assumed to approximate kcal/mol) was used to define hotspots (to account for minor noise around 1.0 kcal/mol, as reflected in the ΔΔ*G* values of known hotspots obtained in the initial analysis on the target crystal structures, see Results).

## RESULTS

In the first part of this study, we report the performance of Rosetta FlexPepDock in CAPRI rounds 28 and 29, the first to include peptide–protein docking challenges. The second part investigates a more general question, that is, how accurate structural models of an interaction need to be to be able to replace or complement experimentally solved structures of complexes. We introduce new measures to enhance the set of current assessment criteria.

### CAPRI performance of Rosetta FlexPepDock

The summary of performance of the Rosetta FlexPepDock team in prediction of the peptide‐protein complex structures in CAPRI rounds 28 and 29 (Targets 60–67) in Table 5 shows that using FlexPepDock, we were able to generate some of the most accurate models submitted by any of the predicting groups: best for T67 (high accuracy for motif, medium accuracy for the full peptide) and right after the Guerois group for T60‐64 (for the full peptide, the Guerois group generated medium accuracy models, while we and the Seok group, generated only acceptable models for 3/5 targets). Thus, we generated some of the best models among all the submissions, in particular for the motif region, when a template structure of the peptide‐protein complex is available (Targets 60–64, 67), but our ability to identify the correct binding site, when it is not known, and consequently to generate accurate models, is still far from complete (Targets 65–66). We shortly detail our protocols and modeling results for the different targets, highlight the successes, and in particular, identify the challenges that need to be addressed next to generate the next generation of improved FlexPepDock modeling tools.

**Table 5:** Performance of the Furman Group Blind Predictions in the CAPRI Peptide–Protein Docking Rounds (Round 28: T60–64; Round 29: T65–67), Using Rosetta FlexPepDock. CAPRI Format Report: # of Acceptable/High***/Medium** Accuracy Models

Target	T67	T60‐64^a^	T60	T61	T62	T63	T64	T65/66
Motif (PPSY; hexamer)	10/6***/4** **high** ^b^	5/4** **medium**	10/8**medium	10/5**medium	10**medium	10acceptable	10/8**medium	–
Full peptide	10/5** **medium**	**3**acceptable	0	0	1acceptable	3acceptable	5acceptable	0

Number of targets for which at least one high***/medium**/acceptable model was submitted.

Top‐ranking results are shown in bold: Rosetta FlexPepDock is the top‐ranking approach for T67, and top‐ranking together with the groups of Guerois and Seok for T60‐64 (see other contributions to this issue).

#### High‐accuracy models of peptide motif‐receptor interactions

##### T67: the importance of selecting a bound structure as template, even if it is from a homolog

**Figure 1:**
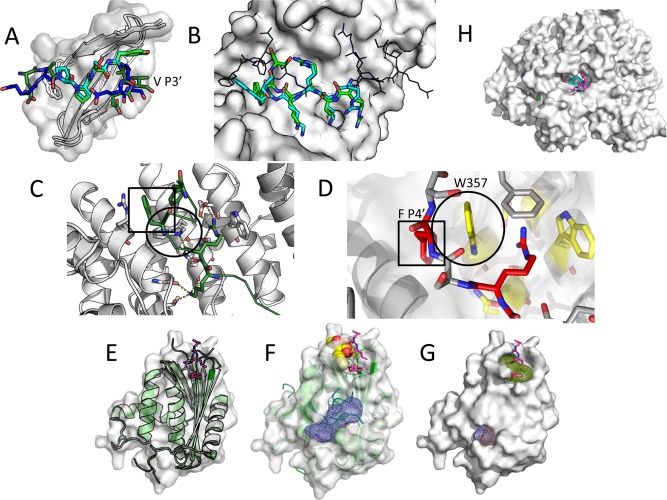
CAPRI performance of the Furman Group: (**A**) T67^41^: ARRDC3 *rpEA* ***PP****S****Y*** *AEVvt* peptide bound to Nedd4 WW3 domain: High accuracy for peptide motif (shown in cyan; *f*nat = 0.83, L_rmsd = 039 Å, I_rmsd = 0.35 Å), medium accuracy for full peptide, including Valine at P _+3’_. Coloring in this and next figures, unless specifically noted: Blue/Cyan—model; Green—crystal structure. (**B–D**) T60‐64^39^: NLS peptides bound to importin minor binding site. (B) Best model of peptide motif (in sticks, *f*nat = 0.83, L_RMSD = 2.42 Å, I_RMSD = 0.79 Å), compared to solved structure of T64. (C,D) Arrangement of aromatic side chains at the interface in the crystal structure (C), and a model (D): In the model, a rotamer flip of the central tryptophan residue (highlighted by a black circle) allows to form a ladder of interface stacking interactions, involving both the receptor and the peptide. This results in incorrect positioning of F P_4’_ (highlighted by a black rectangle). Coloring in (D): yellow–receptor; red–peptide). (**E**–**G**) RNAse bound to SSB‐terminal peptide (T65).^45^ (E) Superposition of the bound conformation and a representative free conformation shows significant movement of an adjacent loop (bound conformation: white and magenta, 4z0u^45^; free conformation: green, 2rn2). (F) Peptimap misses the binding site on the free conformation that we used as template (2rn2), but mapping of SSB‐peptide fragments identifies the site (submission 10; peptide in spheres). (G) Mapping would identify the site and rank it 5th on the bound conformation (yellow mesh; additional predicted sites are on the back, not shown). (**H**) PriA helicase bound to SSB peptide (T66).^46^ Mapping of the full receptor structure using peptimap identifies the binding site and ranks it 5th. However, refinement of the crystal structure does not retain the peptide in the binding site (see Supporting Information Fig. S2), indicating poor resolution of the binding site.

For T67 we were able to generate the model of highest accuracy for the motif, and the most accurate medium accuracy model for the full peptide (Table 5), confirming that FlexPepDock indeed fulfills the goals that it was developed for. Here we describe in short the steps undertaken to generate such a model. The first and crucial step was to select a template for the receptor structure. Because we do not currently model receptor flexibility beyond the side chains, it is important to select an accurate starting template. For T67, the free structure of the Nedd4 WW3 domain was provided, but crystal structures of homolog WW domains bound to peptides were also available (PDB ids 1eg4^50^ and 4lcd^57^). To assess the importance of using the correct protein versus using a bound conformation, we first performed docking simulations on a WW‐peptide interaction where both bound and free receptor structures were available. For the bound run we kept the homolog bound receptor structure, as well as the peptide backbone (PDB id 1eg4), and only changed the peptide sequence by replacing side chains. For the unbound run, we copied that peptide onto the unbound structure provided by the CAPRI organizers. These starting structures were subjected to FlexPepDock local refinement runs. Assuming that the homologous interactions are structurally similar, we expected that the optimization would converge in a narrow funnel centered on a near‐native conformation. The better convergence on the bound receptor conformation (**Supporting Information** Fig. S1) indicates the presence of conformational changes upon binding that are critical for accurate modeling of the peptide. Furthermore, analysis of conformational variation of all solved WW domain structures revealed, despite overall sequence variation, a distinct conformation in the bound structures, mainly of the conserved tryptophan W449 of the WW domain that interacts with the *P*_0’_ proline of the peptide binding motif PP.Y. We therefore chose as a template for the receptor the bound conformation of the dystrophin WW domain (PDB id 1eg4^50^), onto which we threaded the sequence of the nedd4 WW3 domain (using fixed backbone design, see Methods). We added the unaligned part of the peptide in an extended starting conformation and optimized the structure using FlexPepDock *ab initio* and then refinement, under constraints that maintain the bound motif [i.e., constraining the distances between the peptide motif residue P_0'_ and W449 (the conserved tryptophan of the domain), motif residue P P’1 and F438, and between motif residue Y P_4’_ and receptor H442, see Tables 2, 3, 4]. Our best model reproduces the PPXY motif conformation at high resolution. It also positions the specificity determining valine at P _+ 3’_ accurately, but not at atomic resolution [Fig. 1(A) and Table 5].

##### T60‐64: sometimes, simplest is best—Use the homolog template as is and change the sequence to that of the target peptide

Importins contain two distinct sites to bind NLS, and often use both to bind so‐called bi‐partite motifs that contain two repeats of the characteristic basic‐residue‐rich motif. The structural data available prior to the publication of these targets included the complex of such a bi‐partite motif bound to both sites (PDB id 1pjn^58^), as well as the complex of an NLS peptide from SV40TAg that binds specifically to the major site, as evaluated by mutagenesis studies that showed abolishment of binding only when the major site was disrupted (PDB id 1ejl^49^). The latter structure showed that both sites are occupied by the peptide, as turned out to be the case also for the solved structures of T60‐64 upon their release: even though for most of the targets, only mutation in the minor site region of the receptor significantly reduced binding, again, the peptides were found to be bound to both the minor and the major site. In the major site only six peptide residues centered on the motif could be resolved (XKRX[F/W/Y]X), while in the minor site almost all the peptide residues were visible (e.g., *SRG* **QKRSFS** *KAFG for* T60). Assessment of CAPRI targets was done for both sites, and the minor site was assessed both for the hexamer peptide as well as for all the resolved residues. High‐accuracy models were only obtained for hexamers, and primarily for the major site. This site is however of less importance according to mutational studies, indicating that modeling accuracy is not necessarily correlated with model relevance, a topic discussed further below.

For our predictions, we concentrated our efforts on modeling peptides into the *minor* site, as we had identified the targets to contain a minor site‐binding motif (KRX[F/W/Y]XXAF^37^). While our models are among the best submissions for this target, and we were able to generate medium‐accuracy models for the hexamer peptide region [Fig. 1(B)], we (and others) would have done better if we had simply copied the coordinates from the solved structure of the major‐site specific peptide, SV40TAg (bound to the minor groove) and replaced the sequence using threading. The new information provided by the structures of T60‐64 was the formation of an alpha helix c‐terminal to the classical binding motif, which confers specificity to the minor site [Fig. 1 **(C**)]. Unfortunately, we were not able to correctly identify this structure (in fact, only the Guerois group succeeded in this challenge), but instead generated alternative structures with well‐packed arrangements of series of stacked aromatic interactions [Fig. 1 **(D**)]. This over‐rearrangement of receptor side chains, in particular of aromatic rings, is to be addressed in our future peptide docking simulations. We note however, that even with these models we were able to classify the aromatic peptide positions as hotspots, even if it was for the wrong reason. Identification of peptide residue hotspots from inaccurate models is further discussed below.

#### Wrong binding site–wrong prediction

##### T65: The challenge of selecting the right template, and new directions for global peptide docking

For the interaction of bacterial RNAse with the c‐terminal tail of SSB,^45^ no prior information about the binding site was available. We used our solvent mapping‐based PeptiMap protocol to locate peptide‐binding sites on the receptor. But which receptor template structure should we use? Many structural templates were available, differing considerably in a loop region that participates in peptide binding [Fig. 1(E)]. We arbitrarily chose the structure with the best resolution: 2rn2.^59^ PeptiMap missed the binding site on this template [Fig. 1(F)], and therefore, subsequent modeling efforts based on this prediction failed. A template structure with open loop conformation, for example 3aa2,^60^ would have identified the site, but ranked it poorly (data not shown). We note that on the bound structure, PeptiMap would rank the binding site 5th [Fig. 1(G)].

On the peptide side, the same SSB terminal peptide had been solved bound to different receptors, (e.g., PDB id 3sxu^52^). Because conformations of the SSB terminal peptide were known, we decided to proceed in parallel with an alternative approach, in which we docked these peptide conformations instead of solvent molecules (see Methods). With this approach we identified a region adjacent to the correct binding site on 2rn2. Model 10 of our submission to CAPRI is based on this site, but was not accurate enough to pass CAPRI criteria [Fig. 1(G)]. Applying SSB‐peptide mapping to a structure with open loop conformation, for example 3aa2, would clearly have identified the site (model ranked #8; after a cluster of models all localized to the dominant site, which is involved in dimerization in the solved structure 4z0u^45^). This demonstrates that mapping using peptide‐specific conformations can dramatically improve peptide binding site prediction, and in turn, global peptide docking. And indeed, a general implementation of this approach, PeptiDock, shows promising results for global peptide docking, even though subsequent refinement to high resolution is not always straightforward (see Discussion).

The solved structure of SSB peptides bound to other receptors revealed also details about how this peptide binds into a receptor‐binding pocket. In particular, no significant receptor side‐chain rearrangement could be observed, contrasting our models in which we often rearrange aromatic receptor side chains to allow improved packing of peptide aromatic side chains into the pocket [exaggerated repacking of the receptor was also observed in the importin targets; see Fig. 1(D) and Discussion]. Instead, in the crystal structure internal stabilizing interactions in the DIPF’ motif are formed between the buried c‐terminal phenylalanine and the preceding isoleucine that remains exposed. It might be that this hydrophobic residue is further buried by the remaining peptide sequence that was included in the crystallization experiment, but not ordered enough to be resolved. We therefore anticipated that our models would not be accurate, even if the pocket had been known, as our scoring scheme tends to strongly prefer these over‐packed structures, and to penalize exposed hydrophobic side chains.

##### T66: reassessing domain decomposition for PeptiMap peptide binding site mapping

T66 is a complex of PriA helicase, composed of five domains arranged in a circular order, bound to the SSB c‐terminal peptide.^46^ For this target, we miss the binding site, because the current PeptiMap protocol decomposes proteins into distinct domains before mapping each separately for binding sites. If the full receptor surface is mapped, the binding site is identified and ranked 5th [Fig. 1(H)]. This target helped us refine our PeptiMap protocol. The original idea of domain decomposition was to identify binding sites that are contributed by individual domains, since many of our false positive predictions with PeptiMap were due to the identification of sites between two domains, whose relative orientation might be flexible, but the crystal structure presents them as fixed.^15^ In the case of T66, the circular arrangement of the domain into a stable ring suggests that this binding site is rigid, and therefore, domain decomposition is not recommended, as it produces many more suggested binding sites and selection of the correct site is a challenge.

The resolution of the T66 crystal structure is rather low, with poor electron density in the binding site. Indeed, in a standard FlexPepDock refinement run starting from the crystal structure, no convergence is observed, and the peptide moves away (Supporting Information Fig. S2). We therefore would not have expected to succeed in this target, even had we identified the binding site. For this reason, we did not pursue further with T66 in the analysis below.

To summarize, our experience in the peptide docking rounds of CAPRI has been very rewarding: on the one hand, our accurate, top‐performing models for importin and WW domains highlight in a non‐biased way the quality of models that can be obtained from Rosetta FlexPepDock and reinforce our contribution to high‐resolution peptide‐protein modeling. On the other hand, we have identified specific, defined challenges that allow us to focus further development of our peptide docking tools, both for local refinement, as well as for global mapping. New extensions of PeptiMap and FlexPepDock are already under advanced development and we look forward to apply these to the next round of CAPRI peptide docking challenges.

### How accurate does a model need to be to be useful?

We took the opportunity of this CAPRI assessment round of peptide‐protein complexes to study an outstanding question that is often encountered when modeling peptide‐protein interactions: How useful is a model for characterization of interactions? Integral to this investigation is the question of just how accurate—that is, similar in conformation to the solved crystal structure—such a model needs to be to obtain relevant information, and how well this dependency on accuracy is reflected by the current CAPRI quality measures.

In this part, we first define the characteristic features of an interaction from the structure (such as receptor‐peptide hydrogen bonds), as well as from literature (i.e., experimentally determined hot spots), and establish a baseline of performance to detect these features on the solved crystal structure. We then evaluate how well structural models of different quality (as assessed by the current CAPRI criteria, see Table 1) reproduce these baseline features. Analysis of outliers (i.e., models classified as incorrect or acceptable that reproduce a large fraction of native features, as well as models classified as high quality that are less useful for structure‐based characterization of an interface) allows us then to refine our criteria for improved model quality assessment.

#### Definition of critical features of the peptide–protein docking targets of CAPRI

As a first step, we need to define the characteristic features at the peptide‐protein interfaces assessed in this study. Tables 2, 3, 4 list, for the different targets, all interface residues (see Methods for definition) and characteristic features such as hydrogen bonds and salt bridges across the interface. We highlight residues critical for the interaction–peptide motif residues and receptor residues for which experiments have demonstrated considerable decrease in binding upon mutation to alanine or other amino acids. In the following we will term this set of residues “hotspots,” even though for some the observed effect on binding is mild and does not necessarily abolish binding. This constitutes the dataset of interactions and hot spots that we would like to identify in a structure, or in a structural model. We note that for most residues no experimental information is available, including for those that might contribute to binding.

##### The baseline—how well can hotspots be identified from a crystal structure?

An important contribution of a solved structure of a complex is its ability to define interface hotspots that upon mutation will change the interaction affinity and can therefore be used in experiments to further characterize the functional importance of an interaction, without affecting the proteins themselves (e.g., their stability, active site, etc). As mentioned in the introduction, different protocols have been developed to reliably identify these hotspot residues at the interface of a complex structure. How well do they perform on the present set of peptide‐protein complexes, and to what degree do they agree among themselves?

**Figure 2:**
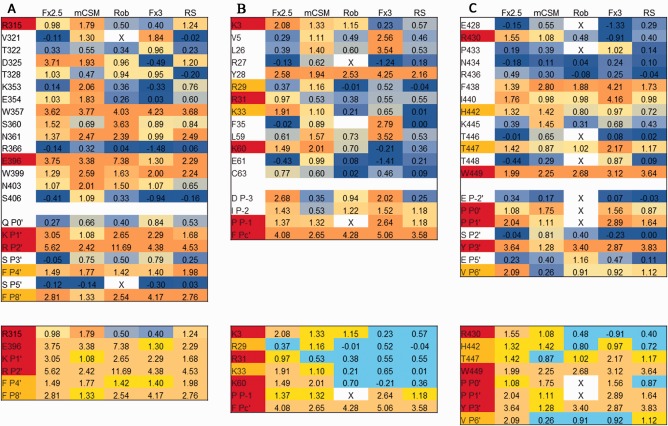
The effect of alanine mutations, as predicted by computational protocols applied to crystal structures. Shown are results for (**A**) T60, (**B**) T65, and (**C**) T67. Upper panels: results for all interface residues, colored from blue to orange for predicted values in the range of [0.00. 2.00] (values beyond are capped to this scale). Lower panels: results for hotspot residues only, colored orange, yellow and cyan for >1.45;>0.95 and the rest. X: value not calculated. Fx: FoldX (versions 2.5 and 3), Rob and RS: Robetta and Rosetta scripts. Residue coloring: Experimentally tested receptor residues that affect binding are colored in red (weaker effect in orange). Previously known peptide motif residues are highlighted in red, and those identified in the studies that report the target structures are in orange. [Color figure can be viewed at wileyonlinelibrary.com]

Figure 2 shows that different protocols paint a rather different picture of a given interaction. Indeed, while predictions of Robetta and FoldX2.5.2 correlate to a certain degree (Spearman correlations of 0.63, p‐val 0.024), no significant correlation is observed between these two protocols and mCSM (0.49 and 0.32, respectively) (Supporting Information Table SI), perhaps reflecting the different basis of energy‐based and structural feature template‐based protocols. Notably, applying the same protocol to different structures of similar peptides bound to the same receptor (T60‐64) shows a more consistent picture, indicating robustness of a given protocol (Supporting Information Fig. S3).

Importantly however, the protocols tend to agree for the residues for which experimental data is available (Fig. 2, lower panels), in particular for the peptide motif residues. All known peptide motif residues are identified by all protocols; and only Valine at P _+ 3’_ which has been shown to play an important role in binding specificity, rather than binding affinity of the WW domain of T67, is not consistently defined as a hotspot (VP _+ 3’_I mutation changes affinity by two‐fold; Tables 2, 3, 4, 41). For importin (T60‐64), all important residues but one are identified by all protocols, but for Bacterial RNAse (T65), the receptor residues involved in binding are often missed.

While the mutation experiments presented in Tables 2, 3, 4 are not always to alanine, the calculated effect is not very different: E396R (T60‐64): predicted ΔΔG = 3.17 versus 3.38, V P _+ 3’_ I (T67): 0.13 versus 0.26, and P P‐1’ S (T65): 1.68 versus 1.32 (as calculated using mCSM). In summary, while predictions of hotspots based on crystal structures vary among different protocols, the known effects are more or less identified by all the protocols. With this in hand, we proceeded to assess how well a model would predict the same effects, compared to a solved crystal structure.

#### Are models useful? How accurate do they need to be?

To assess the contribution of a model to our understanding of an interaction, we assess here three measures that could provide information, building upon the current CAPRI criteria. (1) S‐RMSD—the accuracy of the modeled side chains of residues at the interface. This parameter focuses on the part of the peptide that contributes significantly to specific binding. The most recent CAPRI assessments already calculated this measure, but only the backbone RMSD has been used for classification. We investigate its use for high‐resolution peptide binding assessment (see also Lensink et al. in this issue)^61^; (2) *f*nat_hb_—the fraction of recovered native hydrogen bonds at the interface. This parameter captures how well hydrogen bond networks that often characterize the specific features of binding are recovered in a model compared to a solved crystal structure; (3) Hotspot recovery—the ability of a model to define the residues that are critical for the interaction, both on the receptor and the peptide.

##### Interface side‐chain RMSD as a measure of model accuracy

**Figure 3:**
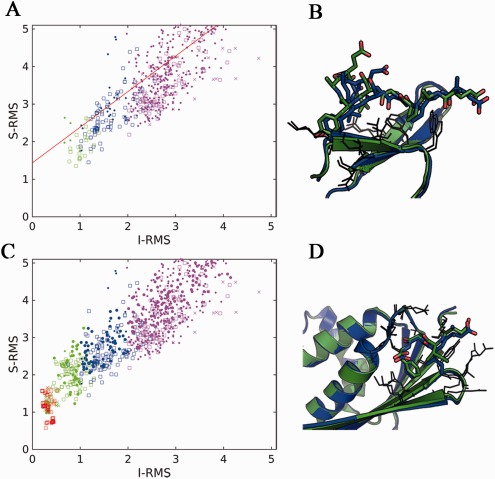
Correlation between different measures of model accuracy. Interface side‐chain RMSD (S‐RMSD) versus interface backbone RMSD (I‐RMSD) for models submitted to CAPRI (regression line in red) (**A**), and models generated by FlexPepDock refinement starting from the native structure (**C**). Models are colored according to CAPRI classification (medium accuracy—green; acceptable model—blue; incorrect models—magenta), and Target: 0 ‐ T60, X‐ T65, and □‐T67. (**B**,**D**) Models suggested for reclassification, based on their accurate side chains (see text for more details; coloring as in Fig. 1). [Color figure can be viewed at wileyonlinelibrary.com]

The modeling accuracy of side chains is highly correlated to that of the backbone (Spearman rank correlation *R* = 0.96; p‐val = 0, see Supporting Information Fig. S4). Zooming in onto the low‐rmsd region (where useful models are located), we can see deviations from the regression line that highlight models defined as acceptable even though their side‐chain RMSD is rather high (up to 5 Å; above the regression line) [Fig. 3(A)]. These occur mainly for the importins, not surprisingly, given the long amino acid residues that form the binding motif, and the fact that the backbone could be modeled based on an available structure. In turn, overall only few models lie significantly below the regression line, and would be judged as incorrect, despite rather low comparative side‐chain RMSD. However, a range of acceptable models for T67 show minimal side‐chain RMSD values, suggesting that these might be reclassified as medium accuracy [see for example T67_P09.M06 in Fig. 3(B)]. We suggest therefore to loosen the threshold for interface and ligand backbone RMSD, in combination with a restriction to the allowed side‐chain RMSD. This would restrict the reclassification to models with well‐modeled side chains. A more robust decision awaits the assessment on a larger set of Targets in the future, which hopefully will also provide more models of better quality. To inspect this higher‐quality regime, we have also looked into a set of models generated by our FlexPepDock refinement protocol starting from the native structures. The distinctly colored steps in the corresponding correlation plot [Fig. 3(C)] indicate that each model quality is characterized by a range of models of minimum, similar side‐chain accuracy, as well as more models of lower accuracy, highlighting the overall robustness of the current classification scheme that at least does not miss any accurate model. Again, this might highlight the need to refine accuracy criteria by including more models with well‐modeled side chains but borderline backbone RMSD values. In particular, the plot also highlights some of the T65 models of sub‐Ångström side‐chain RMSD that are still classified as medium accuracy [see example in Fig. 3(D)]. This indicates that CAPRI criteria might indeed be too stringent in some cases, and inclusion of a combined I‐RMSD and S‐RMSD criterion as suggested here might be the way to generate a refined classification.

##### Recovery of interface characteristics—hydrogen bonds

**Figure 4:**
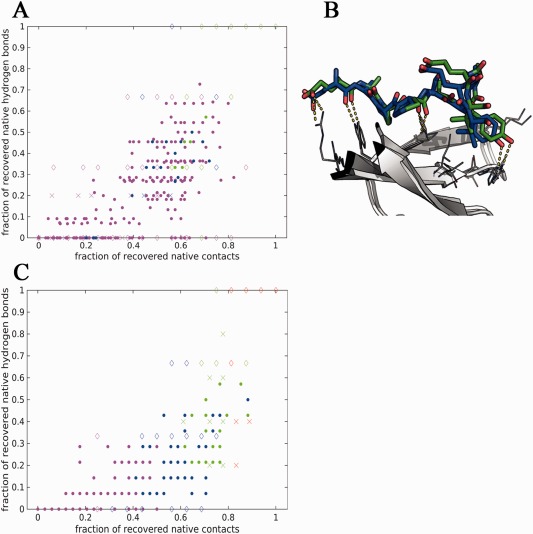
Correlation between different measures of model accuracy. Hydrogen bond recovery (*f*nat_hb_) versus native contact recovery (*f*nat) for models submitted to CAPRI (**A**) and models obtained from local refinement runs (**C**). (**B**) Example of model that recovers all hydrogen bonds, but is ranked as medium due to low native contact recovery (coloring as in Fig. 3). [Color figure can be viewed at wileyonlinelibrary.com]

While CAPRI assesses the ability of a model to identify correct interface contacts, no specific emphasis is put on those that mediate hydrogen bonds, even though these often play a significant role in determining binding affinity and specificity.^62^, ^63^, ^64^ We therefore define a complementary parameter, *f*nat_hb_, to measure hydrogen bond recovery. This parameter shows a good correlation with *f*nat, but also considerable differences, both for the models submitted to CAPRI [Fig. 4(A), spearman correlation r = 0.76, p‐val < 0.001], as well as for local refinement runs [Fig. 4(C), spearman correlation r = 0.8, p‐val < 0.001]. In particular for importins, structures that barely recover any hydrogen bond show a range of native contact recovery. Could hydrogen bond recovery be used as an additional criterion for model quality assessment? Figure 4(B) shows an example model classified as medium (T67_P44.M03: *f*nat = 0.69, L‐RMSD = 1.9 Å, I‐RMSD = 1.29 Å, S‐RMSD = 1.9 Å), where the fact that all hydrogen bonds are recovered may suggest a reassignment to high accuracy model. Overall however, the *f*nat measure is not the main determinant of model classification in this set of targets, and RMSD measures of model accuracy of for example tail regions beyond the hydrogen bond network have a larger impact.

##### Recovery of interface characteristics—interface hotspots

How similar to the crystal structure does a model need to be to detect interface hotspots? We examined two different aspects of hotspots detection. First, we asked how many hotspots does each model identify, and second, we assessed “hit rates” for individual residues at the interface—how often are they suggested hotspots.

**Figure 5:**
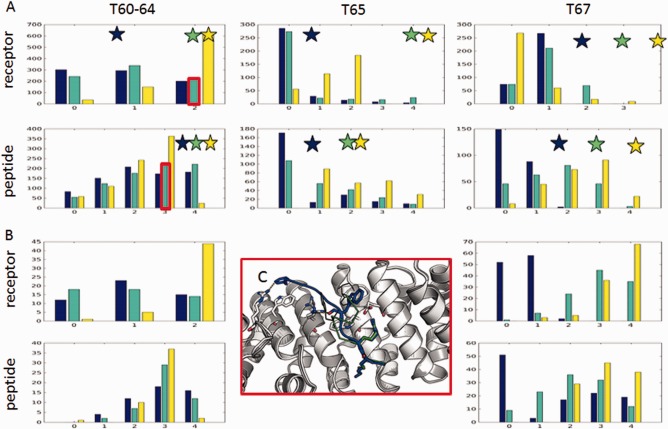
Even incorrect models can identify critical interface receptor and peptide hotspots. (**A**) Distribution of number of hotspots identified by incorrect models. Number of models for which Robetta (blue), FoldX2.5.2 (cyan) or mCSM (yellow) were able to identify a given number of receptor (upper) or peptide (lower row) binding residues for T60‐64 (left), T65 (center) and T67 (right). Stars indicate performance of the corresponding crystal structures. Performance based on crystal structures is highlighted as stars of the same respective color of the protocol. (**B**) Distribution for acceptable and medium accuracy models. (**C**) Example model with high hotspot recovery but poor modeling quality (T62_P36.M04; taken from the boxes outlined in red): While previously known hotspots K_P1’_R_P2’_ are correctly positioned (green and blue overlay), the rest of the peptide extends to form non‐native interactions using F_P4’_, which nevertheless still results in high hotspot recovery rate. [Color figure can be viewed at wileyonlinelibrary.com]

Histograms of how many hotspots are recovered in incorrect and acceptable/medium quality models [Fig. 5(A,B)] show that while as expected most models perform worse as starting point for hotspot residue identification than the crystal structure, even among incorrect models a considerable fraction retrieves as many, or almost as many, hotspots. Thus it would seem that while most models of a large conformational distance from the native structure are unreliable for spotlighting important aspects of a protein–peptide interaction, many of them perform surprisingly well for hotspot identification.

**Figure 6:**
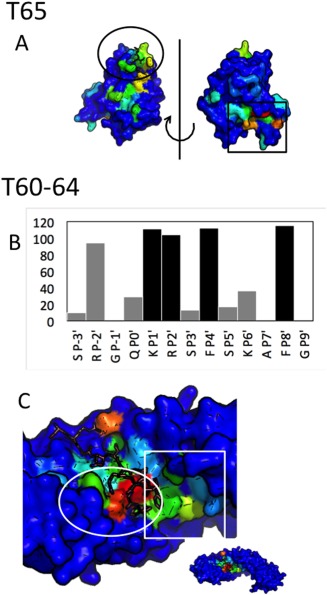
Hitmaps reveal that incorrect models can identify important features of an interaction. Hitmaps show how often each residue on the receptor (and the peptide) is defined as hotspot in the ensemble of incorrect models submitted to CAPRI. (**A**) T65—blind prediction of a binding site: The hitmap of the receptor surface shows a few suggested binding sites, including the SSB binding site (circled), as well as a known dimerization site (square). (**B**,**C**) T60‐64—prediction of secondary motif for minor‐site specific binding: The hitmap recovers previously known, as well as new peptide motif residues (aroP4’ and FP8’) (B), but for the wrong reasons: A wrong binding site is mapped (square), while the pocket binding the FP8’ in the correct orientation is missed (circle) (C). The structures are colored from blue (0) to red (maximum) by the number of times a receptor residue was detected to be important for binding by FoldX2.5.2. Peptide is colored in salmon and represented as sticks. [Color figure can be viewed at wileyonlinelibrary.com]

To further assess this observation, we repeated this analysis at the level of individual residues: For each receptor and peptide residue, we counted how often it was predicted to be a binding hotspot (i.e., a hit). Figure 6(A) shows the structure of the T65 receptor colored according to hit‐rate of receptor hotspots identified in the ensemble of incorrect T65 models. Indeed, while each of these models was classified as incorrect, as an ensemble they map out three possible binding pockets, among them the correct binding pocket for the SSB peptide (in addition to the dominant site which is involved in dimerization in the solved structure, 4z0u,^45^ as mentioned already above). Furthermore, within the correct binding pocket, most hotspots are recovered (except K3 that is located in the flexible n‐terminal tail, data not shown). Thus, when viewed collectively, incorrect models yield a surprising amount of correct information. As for importin, hit‐rate recovers both known and new peptide motif residues [Fig. 6(B)], but not for the right reason: While receptor residues involved in binding of the basic motif are highlighted, hit‐rates locate the pocket to bind FP8’ in a wrong region [Fig. 6(C)]. Finally, for the WW domain, the new region is identified, even if VP’_+3_ is only marginally hit (not shown). We conclude that hotspot recovery can be successful, albeit not necessarily for the correct reason, even if an ensemble of incorrect models is used.

## DISCUSSION AND CONCLUSIONS

Peptide‐protein interactions are now recognized as crucial players in many biological processes, and the modeling of peptide–protein complex structures has come of age. The last CAPRI rounds have included for the first time targets of peptide–motif–receptor interactions, and spurred the development of a range of new approaches to address this particular challenge of the structural modeling of interactions (see also other manuscripts in this issue). Overall, the peptide docking results from these rounds look very promising and indicate a rosy future for the modeling of the many peptide‐mediated interactions of biological relevance.

Here we have summarized successes and challenges of the Furman group submissions that we generated using the Rosetta FlexPepDock protocol, among the first dedicated peptide docking protocols developed.^13^, ^14^ We have been able to create models of top‐ranking accuracy, in particular for the known peptide‐motif regions, but also beyond (T60‐64, T67; Table 5 and Fig. 1). Thus, we have demonstrated here in a blind setting that indeed, in the regime of structural refinement starting from a given template, FlexPepDock is able to produce highly accurate models of an interaction. This was mainly due to the choice of a good receptor template structure: namely, a bound receptor conformation (even if this was a homolog, bound to a different peptide, as in the case of T67). Challenges that we identified include exaggerated receptor side‐chain motility upon binding that is not observed in natural structures of side chains at the interface, but generates well‐scoring, strong inter‐chain stacking interactions [Fig. 1(D)]. This issue can be addressed by defined restriction and improved control of side‐chain moves during optimization. For blind predictions, where the binding site of the peptide was not known (T65&66), we used our PeptiMap protocol to identify possible peptide binding sites on the receptor surface.^15^ Unfortunately, we could not report any success on the blind peptide docking targets. For T66, we identified the reasons for failure as the need for refined rules for separating the structure into independent domain units that are mapped separately. For T65, the challenge was to identify a good template—with an open conformation of the binding site. Thus, while we successfully identified a good template for T67 [Fig. 1(A)], we failed to do so for T65 [Fig. 1(E–G)]. This highlights the need for a more robust protocol for template identification.

On a more general term, our experience in CAPRI rounds 28 and 29 highlighted the need for a better protocol to address global peptide docking, including possible new directions. Several global docking protocols have recently been developed that are able to identify the binding site and generate models of variable accuracy, including the global docking of pre‐folded peptides,^22^, ^24^, ^26^, ^65^, ^66^ as well as the search for conformations similar to existing peptide‐protein interactions.^23^, ^67^ FlexPepDock can in principle serve as the local refinement step of such global docking methods. However, FlexPepDock refinement starting from such models still awaits proper calibration, both to bridge different force fields used, as well as to extend its sampling space so that local minima can be escaped more frequently, in favor of other, nearby minima.

Directly mapping the receptor using peptide fragments (extracted, e.g., from solved structures, as for the SSB c‐terminal peptide) provided encouraging results that did identify the binding site. In a more general setting, these fragments can be extracted from the PDB based on a characterized peptide‐binding motif, as implemented in our recently developed PeptiDock protocol.

Defining a new challenge in CAPRI—here peptide docking—necessitates also the definition of new measures of success. DockQ,^68^ a recently introduced measure of model quality provides one continuous value in the range [0,1], by combining the existing Fnat, L‐RMSD and I‐RMSD measures. This measure is highly correlated with current CAPRI categorization of models, with the advantage of allowing the ranking of models within each category. While such a criterion is superior to a discrete division of models into categories, from our results, it seems that additional parameters, rather than an improved classification based on existing criteria, are needed for better categorization. If the backbone is modeled accurately, it is assumed that the side chains are oriented correctly as well. This is however not necessarily the case, as we have shown here. Thus, our alternative suggestion is to use an additional criterion to assess the model quality, which is not reflected in the present measures, be it as additional measure in the CAPRI scheme, or as part of a combined measure as in DockQ.

We have used this opportunity to ask more generally how accurate a model needs to be to reveal information about the interaction in a similar way as a crystal structure does. While it is clear that for applications such as drug design there is a need for atomic level accuracy, as is reflected by the CAPRI criterion for high accuracy models (Table 1), it seems that for other applications, less accurate models can contribute important information as well, but this depends on the feature we want to recover.

Hydrogen bond networks: As an example, in many interactions, it is the polar interactions, in particular specific hydrogen bonds, which define not only binding affinity, but also binding specificity (e.g.,^69^, ^70^). Therefore, recovery of hydrogen bond networks is a desirable feature of any docking program, in particular for peptide–protein docking. Here we have suggested a new measure, *f*nat_hb_, the fraction of recovered hydrogen bonds (according to definition by HBplus^53^) to assess the hydrogen bond network recovery in models. Our new measure is a subset of the traditional contact measure, *f*nat, which does not distinguish between polar and nonpolar contacts. Therefore, the *f*nat_hb_ measure would provide information particularly important for protein‐peptide binding (Fig. 4).

Side chain modeling accuracy (as measured by S‐RMSD, as already described at the 6th CAPRI evaluation meeting in Tel Aviv, http://www.cs.tau.ac.il/conferences/CAPRI2016) is also a measure that could improve our assessment of models: for peptides in particular, it is important to evaluate how accurately the side chains are positioned, and not only the backbone (I‐RMSD) as has been done until now. Our results suggest that loosening the I‐RMSD (and L‐RMSD) stringency of the CAPRI criteria, together with the introduction of S‐RMSD in the classification, will improve the detection of good models and their separation from the rest [Fig. 3(B,D)].

Identification of interface hot spots is of critical importance for further characterization of an interaction by experiment, and also a good test for the accurate modeling of interface energetics. While this challenging task has not been solved yet, and no general protocol is available that can reliably identify the hotspots in protein complex interfaces, we still could evaluate how well prediction works on models compared to crystal structures. This would give us an indication of how well models can replace tedious experimental work if the goal is to identify binding hotspots. It turns out that for this specific task, the requirements on model accuracy are particularly loose—it is even useful to use different methods for model generation, and combine the ensemble of models that are mostly incorrect to identify peptide residue hotspots—mostly those that are part of the binding motif, as well as hotspots on the receptor. As we show in Figures 2, 5 and 6, even though hotspot recovery can vary considerably depending on the protocol used, and in some cases be rather poor, models can be used often to a similar degree to identify critical residues. The reason for this observation stems from general features that determine binding sites on a protein, which can be detected by mapping of molecules—be it solvents, such as in FTmap approaches^71^ (including PeptiMap^15^), or larger peptides (such as in our new PeptiDock approach, *to be published*). Thus, an approach to detect hotspots based on the mapping of many (incorrect) models would profit from the advantage of pooling information from different, often orthogonal approaches (similar to meta servers that learn to identify e.g., protein interfaces from a pool of interface predictions obtained from other servers^72^). Results from this analysis can also provide a good starting point for local refinement protocols, such as Rosetta FlexPepDock, as described above.

Our study emphasizes the major challenges that we still face in the accurate modeling of the energetics of binding events, which most probably also involve accounting for conformational energetics of the unbound states of the partners. Furthermore, we can also rejoice in the fact that many peptide–protein interactions might be amenable to characterization by modeling, be it at high resolution—as the current CAPRI round definitely proves—or at lower resolution, to identify leads to further experimental characterization of interactions by targeted, model‐driven mutagenesis.

## Supporting information

Supporting Information

## References

[1] Lobb B , Doxey AC. Novel function discovery through sequence and structural data mining. Curr Opin Struct Biol 2016;38:53–61. 27289211 10.1016/j.sbi.2016.05.017

[2] Arkin MR , Tang Y , Wells JA. Small‐molecule inhibitors of protein–protein interactions: progressing toward the reality. Chem Biol 2014;21:1102–1114. 25237857 10.1016/j.chembiol.2014.09.001 PMC4179228

[3] London N , Raveh B , Schueler‐Furman O. Druggable protein–protein interactions–from hot spots to hot segments. Curr Opin Chem Biol 2013;17:952–959. 24183815 10.1016/j.cbpa.2013.10.011

[4] Berman HM , Westbrook J , Feng Z , Gilliland G , Bhat TN , Weissig H , Shindyalov IN , Bourne PE. The protein data bank. Nucleic Acids Res 2000;28:235–242. 10592235 10.1093/nar/28.1.235 PMC102472

[5] Moult J , Fidelis K , Kryshtafovych A , Schwede T , Tramontano A. Critical assessment of methods of protein structure prediction (CASP)—progress and new directions in Round XI. Proteins 2016; Supp 84:4–14. 10.1002/prot.25064 PMC5394799 27171127

[6] Janin J , Henrick K , Moult J , Eyck LT , Sternberg MJ , Vajda S , Vakser I , Wodak SJ. Critical assessment of PI. CAPRI: a critical assessment of predicted interactions. Proteins 2003;52:2–9. 12784359 10.1002/prot.10381

[7] Lensink MF , Wodak SJ. Docking, scoring, and affinity prediction in CAPRI. Proteins 2013;81:2082–2095. 24115211 10.1002/prot.24428

[8] Lensink MF , Wodak SJ. Docking and scoring protein interactions: CAPRI 2009. Proteins 2010;78:3073–3084. 20806235 10.1002/prot.22818

[9] Kilambi KP , Pacella MS , Xu J , Labonte JW , Porter JR , Muthu P , Drew K , Kuroda D , Schueler‐Furman O , Bonneau R , Gray JJ. Extending RosettaDock with water, sugar, and pH for prediction of complex structures and affinities for CAPRI rounds 20–27. Proteins 2013;81:2201–2209. 24123494 10.1002/prot.24425 PMC4037910

[10] Lensink MF , Moal IH , Bates PA , Kastritis PL , Melquiond AS , Karaca E , Schmitz C , van Dijk M , Bonvin AM , Eisenstein M , Jimenez‐Garcia B , Grosdidier S , Solernou A , Perez‐Cano L , Pallara C , Fernandez‐Recio J , Xu J , Muthu P , Praneeth Kilambi K , Gray JJ , Grudinin S , Derevyanko G , Mitchell JC , Wieting J , Kanamori E , Tsuchiya Y , Murakami Y , Sarmiento J , Standley DM , Shirota M , Kinoshita K , Nakamura H , Chavent M , Ritchie DW , Park H , Ko J , Lee H , Seok C , Shen Y , Kozakov D , Vajda S , Kundrotas PJ , Vakser IA , Pierce BG , Hwang H , Vreven T , Weng Z , Buch I , Farkash E , Wolfson HJ , Zacharias M , Qin S , Zhou HX , Huang SY , Zou X , Wojdyla JA , Kleanthous C , Wodak SJ. Blind prediction of interfacial water positions in CAPRI. Proteins 2014;82:620–632. 24155158 10.1002/prot.24439 PMC4582081

[11] Moretti R , Fleishman SJ , Agius R , Torchala M , Bates PA , Kastritis PL , Rodrigues JP , Trellet M , Bonvin AM , Cui M , Rooman M , Gillis D , Dehouck Y , Moal I , Romero‐Durana M , Perez‐Cano L , Pallara C , Jimenez B , Fernandez‐Recio J , Flores S , Pacella M , Praneeth Kilambi K , Gray JJ , Popov P , Grudinin S , Esquivel‐Rodriguez J , Kihara D , Zhao N , Korkin D , Zhu X , Demerdash ON , Mitchell JC , Kanamori E , Tsuchiya Y , Nakamura H , Lee H , Park H , Seok C , Sarmiento J , Liang S , Teraguchi S , Standley DM , Shimoyama H , Terashi G , Takeda‐Shitaka M , Iwadate M , Umeyama H , Beglov D , Hall DR , Kozakov D , Vajda S , Pierce BG , Hwang H , Vreven T , Weng Z , Huang Y , Li H , Yang X , Ji X , Liu S , Xiao Y , Zacharias M , Qin S , Zhou HX , Huang SY , Zou X , Velankar S , Janin J , Wodak SJ , Baker D. Community‐wide evaluation of methods for predicting the effect of mutations on protein–protein interactions. Proteins 2013;81:1980–1987. 23843247 10.1002/prot.24356 PMC4143140

[12] Fleishman SJ , Whitehead TA , Strauch EM , Corn JE , Qin S , Zhou HX , Mitchell JC , Demerdash ON , Takeda‐Shitaka M , Terashi G , Moal IH , Li X , Bates PA , Zacharias M , Park H , Ko JS , Lee H , Seok C , Bourquard T , Bernauer J , Poupon A , Aze J , Soner S , Ovali SK , Ozbek P , Tal NB , Haliloglu T , Hwang H , Vreven T , Pierce BG , Weng Z , Perez‐Cano L , Pons C , Fernandez‐Recio J , Jiang F , Yang F , Gong X , Cao L , Xu X , Liu B , Wang P , Li C , Wang C , Robert CH , Guharoy M , Liu S , Huang Y , Li L , Guo D , Chen Y , Xiao Y , London N , Itzhaki Z , Schueler‐Furman O , Inbar Y , Potapov V , Cohen M , Schreiber G , Tsuchiya Y , Kanamori E , Standley DM , Nakamura H , Kinoshita K , Driggers CM , Hall RG , Morgan JL , Hsu VL , Zhan J , Yang Y , Zhou Y , Kastritis PL , Bonvin AM , Zhang W , Camacho CJ , Kilambi KP , Sircar A , Gray JJ , Ohue M , Uchikoga N , Matsuzaki Y , Ishida T , Akiyama Y , Khashan R , Bush S , Fouches D , Tropsha A , Esquivel‐Rodriguez J , Kihara D , Stranges PB , Jacak R , Kuhlman B , Huang SY , Zou X , Wodak SJ , Janin J , Baker D. Community‐wide assessment of protein–interface modeling suggests improvements to design methodology. J Mol Biol 2011;414:289–302. 22001016 10.1016/j.jmb.2011.09.031 PMC3839241

[13] Raveh B , London N , Schueler‐Furman O. Sub‐angstrom modeling of complexes between flexible peptides and globular proteins. Proteins 2010;78:2029–2040. 20455260 10.1002/prot.22716

[14] Raveh B , London N , Zimmerman L , Schueler‐Furman O. Rosetta FlexPepDock ab‐initio: simultaneous folding, docking and refinement of peptides onto their receptors. PLoS One 2011;6:e18934. 21572516 10.1371/journal.pone.0018934 PMC3084719

[15] Lavi A , Ngan CH , Movshovitz‐Attias D , Bohnuud T , Yueh C , Beglov D , Schueler‐Furman O , Kozakov D. Detection of peptide‐binding sites on protein surfaces: the first step toward the modeling and targeting of peptide‐mediated interactions. Proteins 2013;81:2096–2105. 24123488 10.1002/prot.24422 PMC4183195

[16] Dinkel H , Van Roey K , Michael S , Kumar M , Uyar B , Altenberg B , Milchevskaya V , Schneider M , Kuhn H , Behrendt A , Dahl SL , Damerell V , Diebel S , Kalman S , Klein S , Knudsen AC , Mader C , Merrill S , Staudt A , Thiel V , Welti L , Davey NE , Diella F , Gibson TJ. ELM 2016–data update and new functionality of the eukaryotic linear motif resource. Nucleic Acids Res 2016;44:D294–D300. 26615199 10.1093/nar/gkv1291 PMC4702912

[17] Kozakov D , Brenke R , Comeau SR , Vajda S. PIPER: an FFT‐based protein docking program with pairwise potentials. Proteins 2006;65:392–406. 16933295 10.1002/prot.21117

[18] London N , Raveh B , Schueler‐Furman O. Peptide docking and structure‐based characterization of peptide binding: from knowledge to know‐how. Curr Opin Struct Biol 2013;23:894–902. 24138780 10.1016/j.sbi.2013.07.006

[19] Kilburg D , Gallicchio E. Recent advances in computational models for the study of protein–peptide interactions. Adv Protein Chem Struct Biol 2016;105:27–57. 27567483 10.1016/bs.apcsb.2016.06.002

[20] Antes I. DynaDock: a new molecular dynamics‐based algorithm for protein–peptide docking including receptor flexibility. Proteins 2010;78:1084–1104. 20017216 10.1002/prot.22629

[21] Trellet M , Melquiond AS , Bonvin AM. Information‐driven modeling of protein–peptide complexes. Methods Mol Biol 2015;1268:221–239. 25555727 10.1007/978-1-4939-2285-7_10

[22] Ben‐Shimon A , Niv MY. AnchorDock: blind and flexible anchor‐driven peptide docking. Structure 2015;23:929–940. 25914054 10.1016/j.str.2015.03.010

[23] ee H , Heo L , Lee MS , Seok C. GalaxyPepDock: a protein–peptide docking tool based on interaction similarity and energy optimization. Nucleic Acids Res 2015;43:W431–W435. 25969449 10.1093/nar/gkv495 PMC4489314

[24] Schindler CE , de Vries SJ , Zacharias M. Fully blind peptide–protein docking with pepATTRACT. Structure 2015;23:1507–1515. 26146186 10.1016/j.str.2015.05.021

[25] Zaidman D , Wolfson HJ. PinaColada: peptide‐inhibitor ant colony ad‐hoc design algorithm. Bioinformatics 2016;32:2289–2296. 27153578 10.1093/bioinformatics/btw133

[26] Yan C , Xu X , Zou X. Fully blind docking at the atomic level for protein–peptide complex structure prediction. Structure 2016;24:1842–1853. 27642160 10.1016/j.str.2016.07.021 PMC5080282

[27] Mendez R , Leplae R , De Maria L , Wodak SJ. Assessment of blind predictions of protein–protein interactions: current status of docking methods. Proteins 2003;52:51–67. 12784368 10.1002/prot.10393

[28] Mendez R , Leplae R , Lensink MF , Wodak SJ. Assessment of CAPRI predictions in rounds 3–5 shows progress in docking procedures. Proteins 2005;60:150–169. 15981261 10.1002/prot.20551

[29] Clackson T , Wells JA. A hot spot of binding energy in a hormone‐receptor interface. Science 1995;267:383–386. 7529940 10.1126/science.7529940

[30] Guerois R , Nielsen JE , Serrano L. Predicting changes in the stability of proteins and protein complexes: a study of more than 1000 mutations. J Mol Biol 2002;320:369–387. 12079393 10.1016/S0022-2836(02)00442-4

[31] Benedix A , Becker CM , de Groot BL , Caflisch A , Bockmann RA. Predicting free energy changes using structural ensembles. Nat Methods 2009;6:3–4. 19116609 10.1038/nmeth0109-3

[32] Kortemme T , Baker D. A simple physical model for binding energy hot spots in protein–protein complexes. Proc Natl Acad Sci USA 2002;99:14116–14121. 12381794 10.1073/pnas.202485799 PMC137846

[33] Pires DE , Ascher DB , Blundell TL. mCSM: predicting the effects of mutations in proteins using graph‐based signatures. Bioinformatics 2014;30:335–342. 24281696 10.1093/bioinformatics/btt691 PMC3904523

[34] Geng C , Vangone A , Bonvin AM. Exploring the interplay between experimental methods and the performance of predictors of binding affinity change upon mutations in protein complexes. Protein Eng Des Sel 2016;29:291–299. 27284087 10.1093/protein/gzw020

[35] Potapov V , Cohen M , Schreiber G. Assessing computational methods for predicting protein stability upon mutation: good on average but not in the details. Protein Eng Des Sel 2009;22:553–560. 19561092 10.1093/protein/gzp030

[36] Moal IH , Fernandez‐Recio J. Comment on “protein–protein binding affinity prediction from amino acid sequence.” Bioinformatics 2015;31:614–615. 25381451 10.1093/bioinformatics/btu682

[37] Kosugi S , Hasebe M , Matsumura N , Takashima H , Miyamoto‐Sato E , Tomita M , Yanagawa H. Six classes of nuclear localization signals specific to different binding grooves of importin alpha. J Biol Chem 2009;284:478–485. 19001369 10.1074/jbc.M807017200

[38] Lott K , Cingolani G. The importin beta binding domain as a master regulator of nucleocytoplasmic transport. Biochim Biophys Acta 2011;1813:1578–1592. 21029753 10.1016/j.bbamcr.2010.10.012 PMC3037977

[39] Chang CW , Counago RM , Williams SJ , Boden M , Kobe B. Distinctive conformation of minor site‐specific nuclear localization signals bound to importin‐alpha. Traffic 2013;14:1144–1154. 23910026 10.1111/tra.12098

[40] Leung SW , Harreman MT , Hodel MR , Hodel AE , Corbett AH. Dissection of the karyopherin alpha nuclear localization signal (NLS)‐binding groove: functional requirements for NLS binding. J Biol Chem 2003;278:41947–41953. 12917403 10.1074/jbc.M307162200

[41] Qi S , O'Hayre M , Gutkind JS , Hurley JH. Structural and biochemical basis for ubiquitin ligase recruitment by arrestin‐related domain‐containing protein‐3 (ARRDC3). J Biol Chem 2014;289:4743–4752. 24379409 10.1074/jbc.M113.527473 PMC3931036

[42] Schuchardt BJ , Bhat V , Mikles DC , McDonald CB , Sudol M , Farooq A. Molecular origin of the binding of WWOX tumor suppressor to ErbB4 receptor tyrosine kinase. Biochemistry 2013;52:9223–9236. 24308844 10.1021/bi400987k PMC3906126

[43] Schuchardt BJ , Bhat V , Mikles DC , McDonald CB , Sudol M , Farooq A. Molecular basis of the binding of YAP transcriptional regulator to the ErbB4 receptor tyrosine kinase. Biochimie 2014;101:192–202. 24472438 10.1016/j.biochi.2014.01.011 PMC3995836

[44] McDonald CB , Buffa L , Bar‐Mag T , Salah Z , Bhat V , Mikles DC , Deegan BJ , Seldeen KL , Malhotra A , Sudol M , Aqeilan RI , Nawaz Z , Farooq A. Biophysical basis of the binding of WWOX tumor suppressor to WBP1 and WBP2 adaptors. J Mol Biol 2012;422:58–74. 22634283 10.1016/j.jmb.2012.05.015 PMC3412936

[45] Petzold C , Marceau AH , Miller KH , Marqusee S , Keck JL. Interaction with single‐stranded DNA‐binding protein stimulates *Escherichia coli* ribonuclease HI enzymatic activity. J Biol Chem 2015;290:14626–14636. 25903123 10.1074/jbc.M115.655134 PMC4505529

[46] Bhattacharyya B , George NP , Thurmes TM , Zhou R , Jani N , Wessel SR , Sandler SJ , Ha T , Keck JL. Structural mechanisms of PriA‐mediated DNA replication restart. Proc Natl Acad Sci USA 2014;111:1373–1378. 24379377 10.1073/pnas.1318001111 PMC3910646

[47] Alam N , Zimmerman L , Wolfson NA , Joseph CG , Fierke CA , Schueler‐Furman O. Structure‐based identification of HDAC8 non‐histone substrates. Structure 2016;24:458–468. 26933971 10.1016/j.str.2016.02.002 PMC5590822

[48] London N , Lamphear CL , Hougland JL , Fierke CA , Schueler‐Furman O. Identification of a novel class of farnesylation targets by structure‐based modeling of binding specificity. PLoS Comput Biol 2011;7:e1002170. 21998565 10.1371/journal.pcbi.1002170 PMC3188499

[49] Fontes MR , Teh T , Kobe B. Structural basis of recognition of monopartite and bipartite nuclear localization sequences by mammalian importin‐alpha. J Mol Biol 2000;297:1183–1194. 10764582 10.1006/jmbi.2000.3642

[50] Huang X , Poy F , Zhang R , Joachimiak A , Sudol M , Eck MJ. Structure of a WW domain containing fragment of dystrophin in complex with beta‐dystroglycan. Nat Struct Biol 2000;7:634–638. 10932245 10.1038/77923

[51] Kuhlman B , Baker D. Native protein sequences are close to optimal for their structures. Proc Natl Acad Sci USA 2000;97:10383–10388. 10984534 10.1073/pnas.97.19.10383 PMC27033

[52] Marceau AH , Bahng S , Massoni SC , George NP , Sandler SJ , Marians KJ , Keck JL. Structure of the SSB‐DNA polymerase III interface and its role in DNA replication. EMBO J 2011;30:4236–4247. 21857649 10.1038/emboj.2011.305 PMC3199393

[53] McDonald IK , Thornton JM. Satisfying hydrogen bonding potential in proteins. J Mol Biol 1994;238:777–793. 8182748 10.1006/jmbi.1994.1334

[54] Conchuir SO , Barlow KA , Pache RA , Ollikainen N , Kundert K , O'Meara MJ , Smith CA , Kortemme T. A web resource for standardized benchmark datasets, metrics, and Rosetta protocols for macromolecular modeling and design. PLoS One 2015;10:e0130433. 26335248 10.1371/journal.pone.0130433 PMC4559433

[55] Kellogg EH , Leaver‐Fay A , Baker D. Role of conformational sampling in computing mutation‐induced changes in protein structure and stability. Proteins 2011;79:830–838. 21287615 10.1002/prot.22921 PMC3760476

[56] Van Durme J , Delgado J , Stricher F , Serrano L , Schymkowitz J , Rousseau F. A graphical interface for the FoldX forcefield. Bioinformatics 2011;27:1711–1712. 21505037 10.1093/bioinformatics/btr254

[57] Kamadurai HB , Qiu Y , Deng A , Harrison JS , Macdonald C , Actis M , Rodrigues P , Miller DJ , Souphron J , Lewis SM , Kurinov I , Fujii N , Hammel M , Piper R , Kuhlman B , Schulman BA. Mechanism of ubiquitin ligation and lysine prioritization by a HECT E3. Elife 2013;2:e00828. 23936628 10.7554/eLife.00828 PMC3738095

[58] Fontes MR , Teh T , Jans D , Brinkworth RI , Kobe B. Structural basis for the specificity of bipartite nuclear localization sequence binding by importin‐alpha. J Biol Chem 2003;278:27981–27987. 12695505 10.1074/jbc.M303275200

[59] Katayanagi K , Miyagawa M , Matsushima M , Ishikawa M , Kanaya S , Nakamura H , Ikehara M , Matsuzaki T , Morikawa K. Structural details of ribonuclease H from *Escherichia coli* as refined to an atomic resolution. J Mol Biol 1992;223:1029–1052. 1311386 10.1016/0022-2836(92)90260-q

[60] Tanaka M , Chon H , Angkawidjaja C , Koga Y , Takano K , Kanaya S. Protein core adaptability: crystal structures of the cavity‐filling variants of Escherichia coli RNase HI. Protein Pept Lett 2010;17:1163–1169. 20423323 10.2174/092986610791760342

[61] Lensink MF , Velankar S , Wodak SJ . Modeling protein–protein and protein–peptide complexes: CAPRI 6th edition. Proteins 2017;85:359–377. 27865038 10.1002/prot.25215

[62] Fersht AR , Shi JP , Knill‐Jones J , Lowe DM , Wilkinson AJ , Blow DM , Brick P , Carter P , Waye MM , Winter G. Hydrogen bonding and biological specificity analyzed by protein engineering. Nature 1985;314:235–238. 3845322 10.1038/314235a0

[63] Reichmann D , Rahat O , Albeck S , Meged R , Dym O , Schreiber G. The modular architecture of protein–protein binding interfaces. Proc Natl Acad Sci USA 2005;102:57–62. 15618400 10.1073/pnas.0407280102 PMC544062

[64] Boyken SE , Chen Z , Groves B , Langan RA , Oberdorfer G , Ford A , Gilmore JM , Xu C , DiMaio F , Pereira JH , Sankaran B , Seelig G , Zwart PH , Baker D. De novo design of protein homo‐oligomers with modular hydrogen‐bond network‐mediated specificity. Science 2016;352:680–687. 27151862 10.1126/science.aad8865 PMC5497568

[65] Saladin A , Rey J , Thevenet P , Zacharias M , Moroy G , Tuffery P. PEP‐SiteFinder: a tool for the blind identification of peptide binding sites on protein surfaces. Nucleic Acids Res 2014;42:W221–W226. 24803671 10.1093/nar/gku404 PMC4086095

[66] Kurcinski M , Jamroz M , Blaszczyk M , Kolinski A , Kmiecik S. CABS‐dock web server for the flexible docking of peptides to proteins without prior knowledge of the binding site. Nucleic Acids Res 2015;43:W419–4W424. 25943545 10.1093/nar/gkv456 PMC4489223

[67] Trabuco LG , Lise S , Petsalaki E , Russell RB. PepSite: prediction of peptide‐binding sites from protein surfaces. Nucleic Acids Res 2012;40:W423–W427. 22600738 10.1093/nar/gks398 PMC3394340

[68] Basu S , Wallner B. DockQ: a quality measure for protein–protein docking models. PLoS One 2016;11:e0161879. 27560519 10.1371/journal.pone.0161879 PMC4999177

[69] Joachimiak LA , Kortemme T , Stoddard BL , Baker D. Computational design of a new hydrogen bond network and at least a 300‐fold specificity switch at a protein–protein interface. J Mol Biol 2006;361:195–208. 16831445 10.1016/j.jmb.2006.05.022

[70] Slutzki M , Reshef D , Barak Y , Haimovitz R , Rotem‐Bamberger S , Lamed R , Bayer EA , Schueler‐Furman O. Crucial roles of single residues in binding affinity, specificity, and promiscuity in the cellulosomal cohesin‐dockerin interface. J Biol Chem 2015;290:13654–13666. 25833947 10.1074/jbc.M115.651208 PMC4447945

[71] Kozakov D , Grove LE , Hall DR , Bohnuud T , Mottarella SE , Luo L , Xia B , Beglov D , Vajda S. The FTMap family of web servers for determining and characterizing ligand‐binding hot spots of proteins. Nat Protoc 2015;10:733–755. 25855957 10.1038/nprot.2015.043 PMC4762777

[72] Huang J , Deng R , Wang J , Wu H , Xiong Y , Wang X. metaPIS: a sequence‐based meta‐server for protein interaction site prediction. Protein Pept Lett 2013;20:218–230. 22894160 10.2174/092986613804725208

