# Supplementary material for: FlexPepDock lessons from CAPRI peptide–protein rounds and suggested new criteria for assessment of model quality and utility
Source: Proteins. 2017 Feb 16;85(3):445–62. doi: 10.1002/prot.25230 (PMC6618814; doi:10.1002/prot.25230)

Supplementary material for review

Command line parameters

* Rosetta FlexPepDock prepacking

$FlexPepDocking.{ext} -database ${rosetta_db} -s start.pdb -native native.pdb -flexpep_prepack -ex1 -ex2aro -unboundrot unbound_receptor.pdb

* Rosetta FlexPepDock refinement

$FlexPepDocking.{ext} -database ${rosetta_db} –s start_0001.pdb -native native.pdb - -pep_refine -ex1 -ex2aro -use_input_sc –unboundrot unbound_receptor.pdb -nstruct 100 [-lowres_preoptimize]

* Rosetta FlexPepDock *ab initio* modeling

$FlexPepDocking.{ext} -database ${rosetta_db} -s start_0001.pdb -native native.pdb -out:file:silent decoys.silent -out:file:silent_struct_type binary -lowres_abinitio -pep_refine -ex1 -ex2aro -use_input_sc -unboundrot unbound_receptor.pdb -frag3 <frag3 file> -flexPepDocking:frag5 <frag5 file> -frag9 <frag9 file> -nstruct 50000

* Rosetta fixed backbone design

$fixbb.{ext} -database ${rosetta_db} –s template.pdb -resfile peptide_resfile –ex1-ex2aro –use_input_sc –unboundrot unbound_receptor.pdb -nstruct 1

For more details, see also

www.rosettacommons.org/docs/latest/application_documentation/docking/flex-pep-dock.

Supplementary Tables

Table SI Spearman rank-correlations between the different alanine scanning protocols, on mutations shown in Figure 2. P-values are given in parentheses.

Spearman correlations:

Spearman correlation	Robetta	RosettaScripts	FoldX2.5.2	FoldX3.0	mCSM
Robetta	1	0.85 (0.0004)	0.63 (0.024)	0.73 (0.0063)	0.49 (0.093)
RosettaScripts		1	0.79 (0.002)	0.83 (0.0008)	0.60 (0.034)
FoldX2.5.2			1	0.57 (0.0449)	0.32 (0.28)
FoldX3.0				1	0.58 (0.0425)
mCSM					1

Supplementary Figures

Supplementary Figure 1: Stronger convergence in a peptide docking simulation on a bound receptor conformation of a homologous WW domain (dystrophin, PDB id 1eg4[37]) **(A)** compared to the unbound structure template provided for **T67 (B)**.

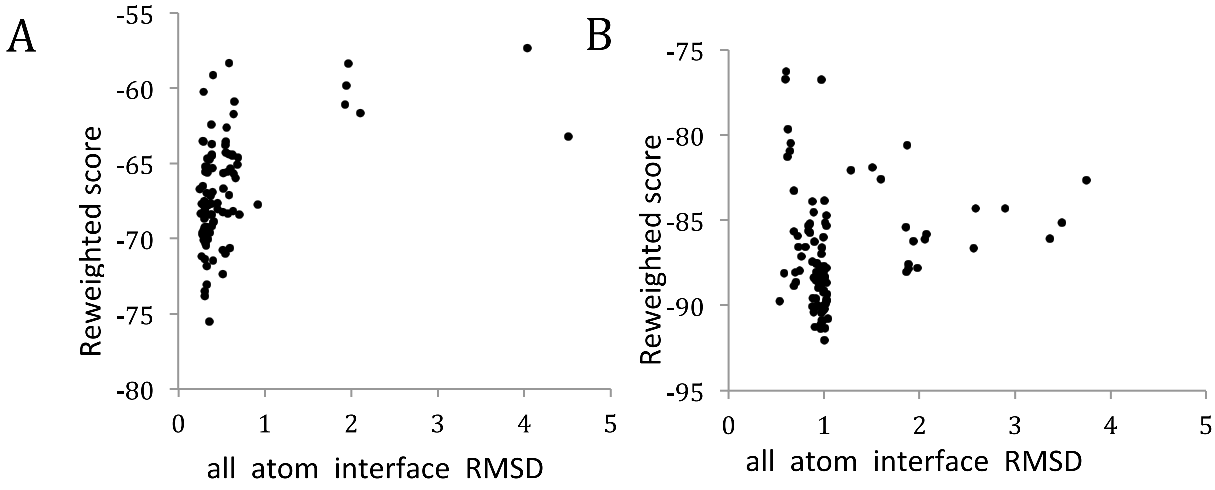

Supplementary Figure 2:

Left: A local FlexPepDock refinement run starting from the solved structure of T66 results in considerable divergence, indicating that even if starting from a nearby structure, an accurate model would not be created. This might also indicate a poorly-resolved structure, as also supported by poor electron density in the region of the binding pocket. **Right:** For comparison, a corresponding local refinement run for T65 converges to a funnel within 0.5 Å RMSD.

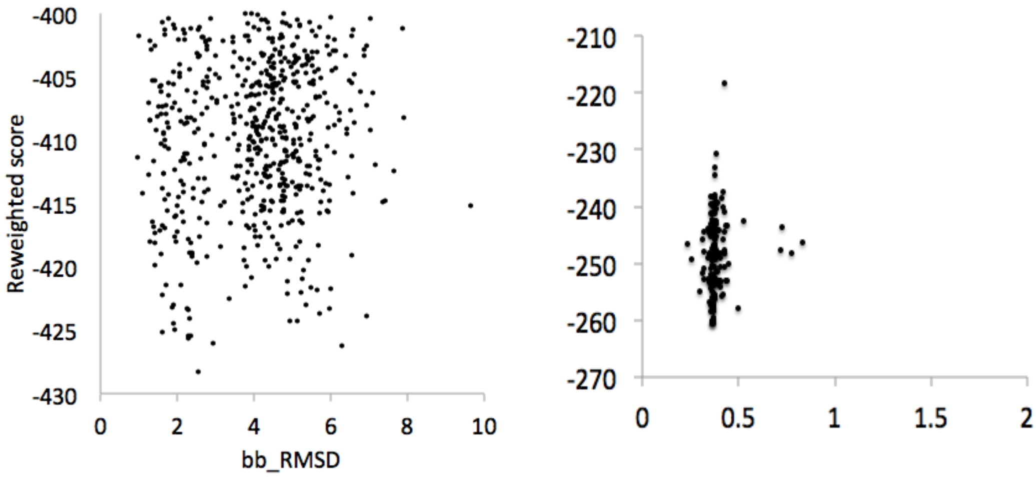

T66 **T65**

Supplementary Figure S3: Computational alanine scanning produces similar results using different structures of the same interaction. Representative results are shown for FoldX2.5 applied to T60-T64 (see also **Figure 2**).

Position	T60	T61	T62	T63	T64
R315A	0.98	0.42	0.52	0.98	0.45
N319A	0.06	0.15	0.08	0.10	0.17
V321A	-0.11	-0.11	-0.25	-0.09	-0.22
T322A	0.33	0.36	0.39	1.33	1.40
D325A	3.71	1.16	1.21	1.65	1.30
T328A	1.03	0.70	1.23	1.04	1.06
K353A	0.14	0.04	-0.03	0.23	0.02
E354A	1.03	1.27	0.74	0.63	0.54
W357A	3.62	3.44	3.57	3.59	3.71
S360A	1.52	1.44	1.62	0.93	1.55
N361A	1.37	1.39	1.64	1.35	1.61
K392A	0.12	0.31	0.31	0.25	0.08
K395A	0.09	0.02	0.03	0.08	-0.02
E396A	3.75	3.31	2.91	1.36	3.09
W399A	1.29	1.02	0.99	0.98	1.03
N403A	1.07	X	0.43	0.21	1.14
S406A	-0.41	-0.34	-0.38	-0.26	0.22
Q/R4A	0.27	0.38	0.92	0.45	0.71
K5A	3.05	2.90	3.67	2.83	3.16
R6A	5.62	5.19	5.25	3.26	4.95
7A	-0.05	0.00	0.06	1.04	1.27
Aro8A	1.49	1.25	0.66	1.35	0.90
S/R9A	-0.12	-0.18	-1.69	-0.22	-0.31
F12A	2.81	2.05	2.15	1.84	2.33

Supplementary Figure S4: Full plot of correlation between different measures of model accuracy – S-RMSD (side chain) *vs.* l-RMSD (backbone) RMSD for models submitted to CAPRI.

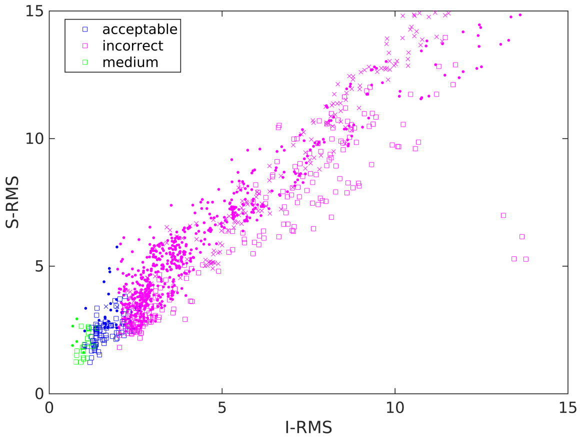

Supplement: Supplementary file 1 — Supporting Information [file PROT-85-445-s001.pdf]
